# Phase 2 trial of PSMA PET CT versus planar bone scan and CT in prostate cancer patients progressing while on androgen deprivation therapy

**DOI:** 10.1038/s41598-024-75589-6

**Published:** 2024-10-18

**Authors:** John Nikitas, Andrei Gafita, Matthias R. Benz, Loïc Djaïleb, Andrea Farolfi, Masatoshi Hotta, Ida Sonni, Rejah Alano, Matthew Rettig, John Shen, Wesley Armstrong, Tristan Grogan, Sandy Liu, Johannes Czernin, Jeremie Calais

**Affiliations:** 1 Department of Radiation Oncology, University of California, Los Angeles, CA USA; 2 Division of Nuclear Medicine and Molecular Imaging, Department of Radiology and Radiological Science, Johns Hopkins University School of Medicine, Baltimore, MD USA; 3 Ahmanson Translational Theranostics Division, Department of Molecular and Medical Pharmacology, University of California, Los Angeles, CA USA; 4 Nuclear Medicine, IRCCS Azienda Ospedaliero-Universitaria di Bologna, Bologna, Italy; 5 Department of Radiological Sciences, University of California, Los Angeles, CA USA; 6 Department of Experimental and Clinical Medicine, University Magna Graecia, Catanzaro, Italy; 7 Departments of Medicine and Urology, University of California, Los Angeles, CA USA; 8 Hematology-Oncology Section, Medicine Service, Greater Los Angeles Veterans Affairs Healthcare System, Los Angeles, CA USA; 9 Department of Medicine Statistics Core, University of California, Los Angeles, CA USA; 10 Department of Medical Oncology & Therapeutics Research, City of Hope, Los Angeles, CA USA

**Keywords:** Androgen deprivation therapy, Biochemical recurrence, Bone metastases, Bone scan, Prostate-specific membrane antigen PET/CT, Prostate cancer, Bone cancer, Clinical trials, Cancer imaging, Bone imaging, Radionuclide imaging, Whole body imaging

## Abstract

For prostate cancer patients who experience biochemical progression during androgen deprivation therapy (ADT), prostate-specific membrane antigen positron emission tomography/computed tomography (PSMA PET/CT) has not been prospectively compared to planar bone scan plus CT. This was a single-arm, head-to-head, prospective phase II trial (NCT04928820) designed to enroll 102 men with prostate cancer who experienced biochemical progression (rising prostate-specific antigen [PSA] ≥ 1 ng/mL) during ADT. All patients received 68Ga-PSMA-11 PET/CT and 99mTc-MDP planar bone scans. Each scan was interpreted by three central independent readers. The primary endpoint was the per-patient bone metastasis detection rate of PSMA PET/CT versus planar bone scan and CT. Secondary endpoints compared the number of bone metastases detected per patient and the inter-reader agreement of each imaging modality. Twenty-two men were enrolled between July 2021 and June 2022. Due to slow accrual following approval of PSMA PET radiotracers in the U.S. and a lack of a statistical signal between the two imaging modalities on interim analysis, this trial was closed early on October 2022. Median PSA was 8.5 ng/mL (interquartile range: 1.6–77.6). There was 100% agreement between the two scans. Six patients (27%) had negative findings and 16 patients (73%) had positive findings on both scans. PSMA PET/CT and bone scan plus CT detected an equal number of bone lesions for 14 patients (64%), PSMA PET/CT detected more bone lesions for six patients (27%), and bone scan plus CT detected more bone lesions for two patients (9.1%) (p = 0.092). The inter-reader agreement rates of PSMA PET/CT and bone scan plus CT were 96% and 82%, respectively (*p* = 0.25). In men with biochemical progression during ADT, 68Ga-PSMA-11 PET/CT and 99mTc-MDP planar bone scan plus CT had identical bone metastasis detection rates. Bone scan plus CT can continue to serve as a cost-effective and readily accessible restaging modality in patients with biochemical progression. ClinicalTrials.gov NCT04928820. Registered 16/06/2021.

**Supplementary Information:**

The online version contains supplementary material available at 10.1038/s41598-024-75589-6.

## Introduction

Prostate-specific membrane antigen positron emission tomography/computed tomography (PSMA PET/CT) offers superior accuracy for detecting non-localized prostate cancer at initial diagnosis compared to bone scan and CT^1,2^. Early studies suggest that PSMA PET/CT also has high detection rates of metastatic disease in patients with biochemical progression during androgen deprivation therapy (ADT)^3,4^. However, prospective data comparing the efficacy of PSMA PET/CT versus bone scan plus CT in this setting are not available and guidelines from the National Comprehensive Cancer Network do not favor one imaging modality over the other^5^.

Upstaging in this patient population can lead to significant changes in systemic therapy management, including adding or changing androgen receptor pathway inhibitors (ARPIs)^6,7^, chemotherapies^8,9^, or radioligand therapies^10^. This raises the question of whether conventional imaging modalities like bone scan plus CT should continue to play an important role in this setting.

We aimed to prospectively compare the bone metastasis detection rates of ^68^Ga-PSMA-11 PET/CT and ^99m^Tc-MDP planar bone scan plus CT in the setting of biochemical progression during ADT.

## Materials and methods

### Study population

This was a self-funded, single-center, single-arm, prospective phase II trial of head-to-head imaging comparison (ClinicalTrials.gov identifier: NCT04928820). The institutional review board approved this study (IRB#21–000102) and all subjects signed written, informed consent. The full study protocol is detailed in the Supplementary Material.

This study enrolled men with prostate cancer who experienced biochemical progression (two consecutive rises in prostate-specific antigen [PSA] ≥ 1 week apart) during ADT and had serum PSA ≥ 1 ng/mL. All patients received ^68^Ga-PSMA-11 PET/CT and ^99m^Tc-MDP planar bone scans within 30 days of each other. Initiation of a new therapy was not allowed between scans. Three blinded independent central readers interpreted each scan to decide on the presence and number (0, 1, 2, 3, 4, 5, 6–10, > 10, or diffuse) of bone metastases. For the PSMA PET/CT, readers separately evaluated whether there were extra-osseous lesions in the prostate bed, pelvic lymph nodes, extra-pelvic lymph nodes, and viscera. PSMA PET/CT readers also evaluated whether there were osseous and extra-osseous lesions visible on CT alone. Lesions were considered positive on CT if they were ≥ 10 mm for the prostate bed, bone, and viscera and ≥ 15 mm for pelvic and extra-pelvic lymph nodes. MB, LD, and AF interpreted each PSMA PET/CT and JCa, MH, and IS interpreted each ^99m^Tc-MDP planar bone scan. Readers were board-certified nuclear medicine physicians and were provided with guidelines and case report forms (Supplemental Material). Majority rule was used for cases with inter-reader disagreement.

### Study endpoints

The primary endpoint was the per-patient detection rate of bone metastasis (0 versus ≥ 1 lesion). McNemar’s test was used to compare the detection rates of PSMA PET/CT versus planar bone scan plus the CT portion of the PET/CT. The study was designed to enroll 102 patients to have 80% power to detect a 12% difference between PSMA PET/CT and bone scan plus CT (hypothesized detection rates were 35% versus 23%, respectively) (Supplementary Table 1).

The number of bone metastases detected per patient on PSMA PET/CT and bone scan plus CT were compared using Wilcoxon signed-rank test. The per-patient inter-reader agreement rates of detecting bone metastasis on PSMA PET/CT and bone scan plus CT were compared using McNemar’s test. The median PSA levels of patients with positive and negative scans were compared using the Mann-Whitney U test. The detection rates of extra-osseous metastasis on PSMA PET/CT and CT alone were compared using McNemar’s test. The per-patient inter-reader agreement rates of detecting extra-osseous metastasis on PSMA PET/CT and CT alone were compared using McNemar’s test. *P* < 0.05 was considered statistically significant. All statistical analyses were performed using SPSS Statistics, version 28 (IBM Corp.).

## Results

This study was terminated prematurely following U.S. Food and Drug Administration approval of PSMA radiotracers in 2022. This led to widespread insurance coverage of PSMA PET/CTs for biochemical progression, significantly reducing this trial’s accrual rate. Furthermore, interim analysis after the first 21 patients enrolled in the trial found 100% (95% confidence interval, 81–100%) agreement between the two scans, which exceeded the hypothesized rate of 81%. Due to poor accrual and lack of a statistical signal, this trial was closed prematurely on October 2022.

###  Study population

**Figure 1:**
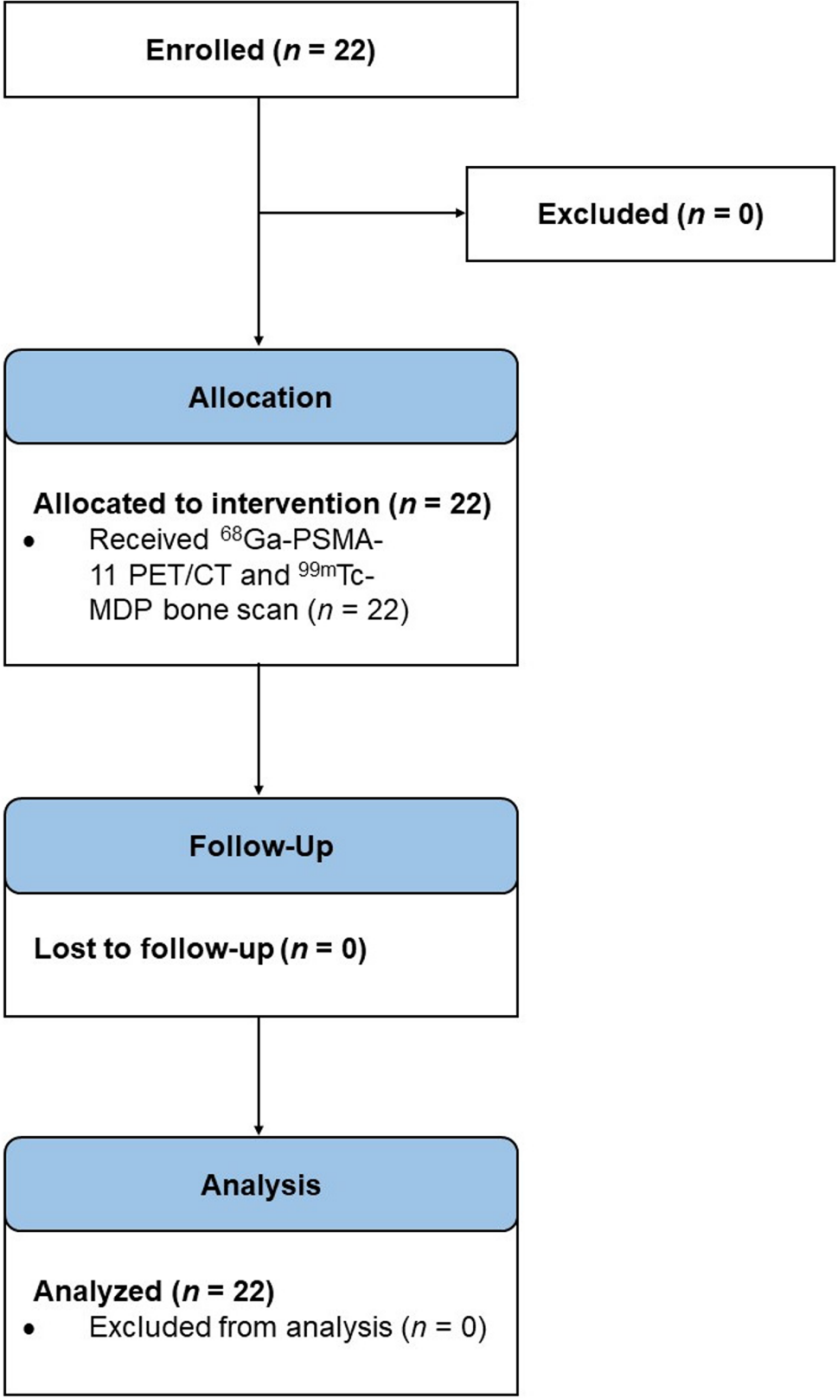
Flow diagram.

Twenty-two patients were enrolled between July 2021 and June 2022 (Fig. 1). Median age was 71 years (interquartile range [IQR]: 65–76 years) (Table 1). Median PSA was 8.5 ng/mL (IQR: 1.6–77.6 ng/mL). Median time from initial diagnosis was 5.6 years (IQR: 2.2–10.4 years). Seven patients (32%) had prior radical prostatectomy, 13 (59%) had prior prostate-directed radiotherapy, and 8 (36%) had prior chemotherapy. Median time between scans was 13.5 days (IQR: 5.0-24.5 days). Seventeen men (77%) underwent bone scan prior to PSMA PET/CT. Five patients (23%) were on ADT, 10 (45%) were on ADT plus ARPI, six (27%) were on ADT plus chemotherapy, and one (4.5%) was on ADT plus olaparib.

**Table 1:** Patient characteristics.

	All patients (*n* = 22)
**Median age**	70.5 years (IQR: 65.8–76.0 years)
**Median PSA**	8.5 ng/mL (IQR: 1.6–77.6 ng/mL)
**Median time between scans**	13.5 days (IQR: 5.0-24.5 days)
**Current therapy**	
ADT	5 (23%)
ADT + ARPI	10 (45%)
ADT + chemotherapy	6 (27%)
ADT + olaparib	1 (4.5%)
**Median time from initial diagnosis**	5.6 years (IQR: 2.2–10.4 years)
**Initial biopsy ISUP grade group**	
2	2 (9.1%)
3	1 (4.5%)
4	5 (23%)
5	8 (36%)
Unknown	6 (27%)
**Initial NCCN risk group**	
Favorable intermediate-risk	2 (9.1%)
Unfavorable intermediate-risk	1 (4.5%)
High-risk	4 (18%)
Very high-risk	3 (14%)
Regional	2 (9.1%)
Metastatic	7 (32%)
Unknown	3 (14%)
**Prior therapies**	
RP + RT + ADT + ARPI	2 (9.1%)
RP + RT + ADT + ARPI + chemotherapy	5 (23%)
RT + ADT	2 (9.1%)
RT + ADT + ARPI	4 (18%)
ADT	1 (4.5%)
ADT + ARPI	4 (18%)
ADT + ARPI + olaparib	1 (4.5%)
ADT + ARPI + chemotherapy	3 (14%)

Abbreviations: IQR, interquartile range; PSA, prostate-specific antigen; ADT, androgen deprivation therapy; ARPI, androgen receptor pathway inhibitor; ISUP, International Society of Urological Pathology; NCCN, National Comprehensive Cancer Network; RP, radical prostatectomy; RT, radiotherapy.

### Bone metastasis findings

**Figure 2:**
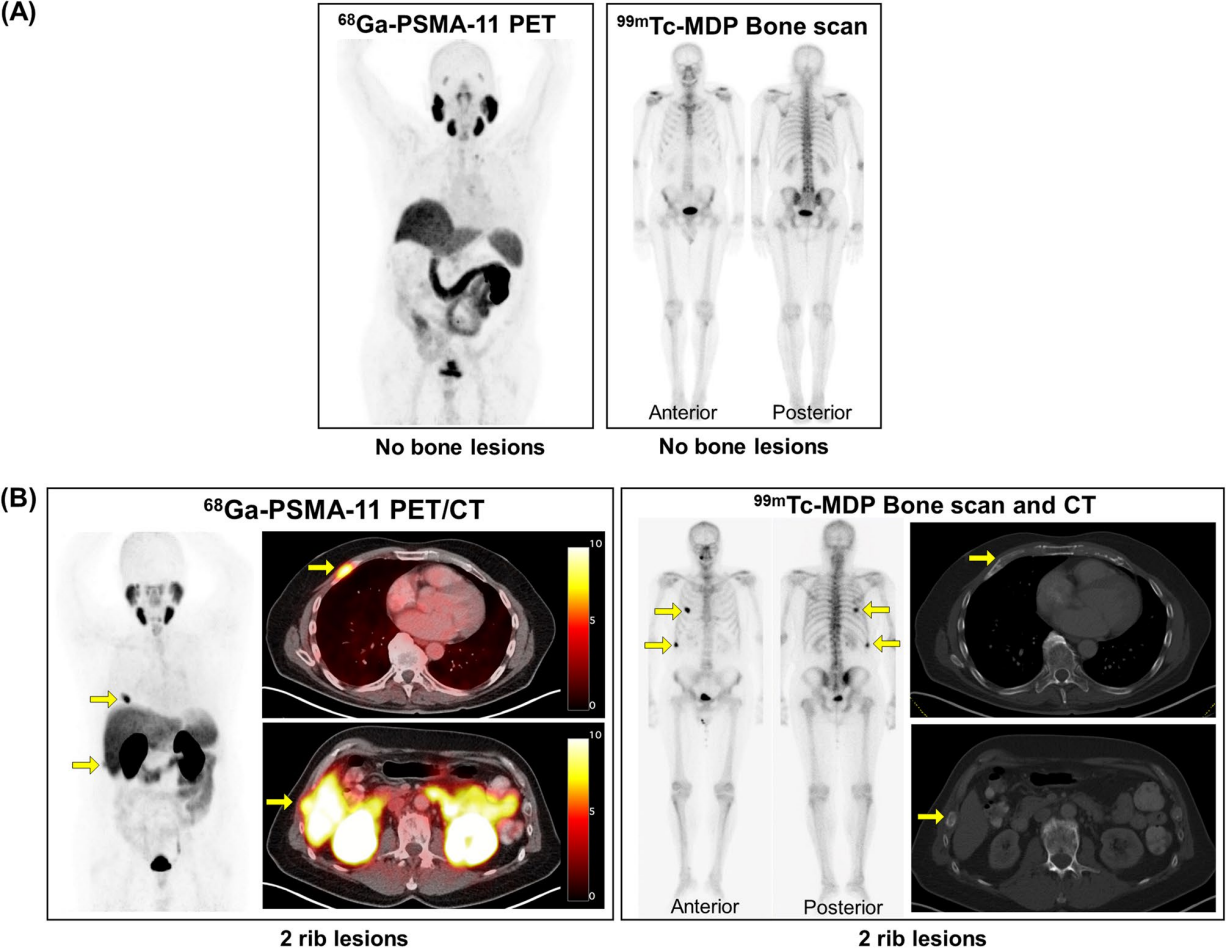
(**A**)Patient 1 is a 72-year-old male with Gleason 5+5 prostate cancer who completed prostate radiotherapy and had a rising PSA (75 ng/mL) while continuing androgen deprivation therapy (ADT) and enzalutamide. There were no detected bone lesions on PSMA PET/CT or ^99m^Tc-MDPplanar bone scan. (**B**) Patient 18 is a 70-year-old male with *de novo* metastatic prostate cancer who started ADT with abiraterone acetate and had a rising PSA (1.4 ng/mL) with two concordant rib lesions on PSMA PET/CT and ^99m^Tc-MDPplanar bone scan plus CT.

Per majority rule applied centrally on the blinded reads, six patients (27%) had no detected bone lesions on PSMA PET/CT or bone scan plus CT and 16 patients (73%) had bone lesions on PSMA PET/CT and bone scan plus CT (Tables 2, 3 and 4). Both scans had identical per-patient detection rates (*p* = 1.0). Figure 2 shows examples of negative and positive PSMA PET/CT and bone scans.

**Table 2:** Contingency table of the consensus majority reads per patient (*n* = 22 patients) comparing bone metastasis detection rates of ^68^Ga-PSMA PET/CT and ^99m^Tc-MDP bone scan plus CT.

	^99m^Tc-MDP Bone Scan plus CT +	^99m^Tc-MDP Bone Scan plus CT -
^**68**^**Ga-PSMA PET/CT+**	73%	0%
^**68**^**Ga-PSMA PET/CT-**	0%	27%

Abbreviations: PSMA PET/CT, prostate specific membrane antigen positron emission tomography/computed tomography.

**Table 3:** PSMA PET/CT and ^99m^Tc-MDP planar bone scan results.

Patient No.	Presence of bone metastasis		Number of bone lesions		Extra-osseous disease on PSMA PET/CT				Extra-osseous disease on CT alone			
	PSMA PET/CT	Bone Scan/CT	PSMA PET/CT	Bone Scan/CT	T+ disease	N+ disease	M1a disease	M1c disease	T+ disease	N+ disease	M1a disease	M1c disease
**1**	No	No	0	0	**Yes**	No	**Yes**	No	No	No	**Yes**	No
**2**	No	No	0	0	**Yes**	No	No	No	No	No	No	No
**3**	**Yes**	**Yes**	>10	3	No	No	No	No	No	No	No	No
**4**	**Yes**	**Yes**	>10	3	No	No	**Yes**	No	No	No	No	No
**5**	**Yes**	**Yes**	Diffuse	Diffuse	No	**Yes**	**Yes**	No	No	No	No	No
**6**	No	No	0	0	**Yes**	No	No	No	No	No	No	No
**7**	**Yes**	**Yes**	>10	>10	No	No	No	No	No	No	No	No
**8**	No	No	0	0	**Yes**	**Yes**	No	No	**Yes**	**Yes**	No	No
**9**	**Yes**	**Yes**	Diffuse	Diffuse	No	No	No	**Yes**	No	No	No	No
**10**	**Yes**	**Yes**	5	2	No	No	No	No	No	No	No	No
**11**	**Yes**	**Yes**	2	1	No	No	No	No	No	No	No	No
**12**	**Yes**	**Yes**	>10	>10	No	No	No	No	No	No	No	No
**13**	**Yes**	**Yes**	>10	>10	No	No	No	No	No	No	No	No
**14**	No	No	0	0	No	No	No	No	No	No	No	No
**15**	No	No	0	0	**Yes**	No	No	No	No	No	No	No
**16**	**Yes**	**Yes**	2	5	**Yes**	**Yes**	No	No	**Yes**	No	No	No
**17**	**Yes**	**Yes**	3	4	**Yes**	No	No	No	No	No	No	No
**18**	**Yes**	**Yes**	2	2	No	No	No	No	No	No	No	No
**19**	**Yes**	**Yes**	Diffuse	Diffuse	**Yes**	No	No	No	**Yes**	No	No	No
**20**	**Yes**	**Yes**	>10	1	No	No	No	No	No	No	No	No
**21**	**Yes**	**Yes**	>10	>10	No	**Yes**	No	No	No	**Yes**	No	No
**22**	**Yes**	**Yes**	5	3	**Yes**	**Yes**	**Yes**	**Yes**	No	**Yes**	**Yes**	**Yes**
**Total**	**16/22**	**16/22**	**-**	**-**	**9/22**	**5/22**	**4/22**	**2/22**	**3/22**	**3/22**	**2/22**	**1/22**

Abbreviations: PSMA PET/CT, prostate-specific membrane antigen positron emission tomography/computed tomography.

**Table 4:** Per-patient reads of the six independent central readers (n = 22 patients) evaluating the presence of bone lesions.

Patient No.	Presence of Bone Lesions on^ 68^Ga-PSMA PET/CT				Presence of Bone Lesions on ^99m^Tc-MDP Bone Scan plus CT			
	Reader 1	Reader 2	Reader 3	Inter-reader agreement on the presence of bone metastasis	Reader 4	Reader 5	Reader 6	Inter-reader agreement on the presence of bone metastasis
**1**	No	No	No	**Yes**	No	**Yes**	No	No
**2**	No	No	No	**Yes**	No	No	No	**Yes**
**3**	**Yes**	**Yes**	**Yes**	**Yes**	**Yes**	**Yes**	**Yes**	**Yes**
**4**	**Yes**	**Yes**	**Yes**	**Yes**	**Yes**	**Yes**	**Yes**	**Yes**
**5**	**Yes**	**Yes**	**Yes**	**Yes**	**Yes**	**Yes**	**Yes**	**Yes**
**6**	No	No	No	**Yes**	No	No	No	**Yes**
**7**	**Yes**	**Yes**	**Yes**	**Yes**	**Yes**	**Yes**	**Yes**	**Yes**
**8**	No	No	No	**Yes**	No	No	No	**Yes**
**9**	**Yes**	**Yes**	**Yes**	**Yes**	**Yes**	**Yes**	**Yes**	**Yes**
**10**	**Yes**	No	**Yes**	No	**Yes**	No	**Yes**	No
**11**	**Yes**	**Yes**	**Yes**	**Yes**	**Yes**	**Yes**	**Yes**	**Yes**
**12**	**Yes**	**Yes**	**Yes**	**Yes**	**Yes**	**Yes**	**Yes**	**Yes**
**13**	**Yes**	**Yes**	**Yes**	**Yes**	**Yes**	**Yes**	**Yes**	**Yes**
**14**	No	No	No	**Yes**	No	No	No	**Yes**
**15**	No	No	No	**Yes**	No	No	No	**Yes**
**16**	**Yes**	**Yes**	**Yes**	**Yes**	**Yes**	**Yes**	**Yes**	**Yes**
**17**	**Yes**	**Yes**	**Yes**	**Yes**	**Yes**	**Yes**	**Yes**	**Yes**
**18**	**Yes**	**Yes**	**Yes**	**Yes**	**Yes**	**Yes**	**Yes**	**Yes**
**19**	**Yes**	**Yes**	**Yes**	**Yes**	**Yes**	**Yes**	**Yes**	**Yes**
**20**	**Yes**	**Yes**	**Yes**	**Yes**	No	**Yes**	**Yes**	No
**21**	**Yes**	**Yes**	**Yes**	**Yes**	**Yes**	**Yes**	**Yes**	**Yes**
**22**	**Yes**	**Yes**	**Yes**	**Yes**	**Yes**	No	**Yes**	No
**Total**	**16/22**	**15/22**	**16/22**	**21/22**	**15/22**	**15/22**	**16/22**	**18/22**

Abbreviations: PSMA PET/CT, prostate-specific membrane antigen positron emission tomography/computed tomography.

PSMA PET/CT and bone scan plus CT detected an equal number of bone lesions for 14 patients (64%). PSMA PET/CT detected more bone lesions for six patients (27%), and bone scan plus CT detected more bone lesions for two patients (9.1%). There was no statistically significant difference between the two imaging modalities in the number of detected bone lesions per patient (*p* = 0.09).

There was no significant difference in inter-reader agreement between PET/CT and bone scan plus CT (96% versus 82%, *p* = 0.25) (Tables 3 and 4). Kappa coefficients for inter-reader comparisons were 0.89-1 for PSMA PET/CT and 0.58–0.89 for bone scan plus CT (Supplemental Table 2). There was no significant difference in median PSA between patients with positive and negative scans (8.50 versus 9.15 ng/mL, *p* = 0.45).

### Extra-osseous metastasis findings

Thirteen patients (59%) had extra-osseous metastasis on PSMA PET/CT (Table 3; Supplemental Table 3). Nine patients (41%) had disease in the prostate or prostate bed, five (23%) had pelvic nodal metastasis, four (18%) had extra-pelvic nodal metastasis, and two (9.1%) had visceral metastasis.

Six patients (27.3%) had extra-osseous metastasis on the CT portion of the PET/CT (Table 3; Supplemental Table 4). Three patients (13.6%) had disease in the prostate or prostate bed, three patients (13.6%) had pelvic nodal metastasis, two patients (9.1%) had extra-pelvic nodal metastasis, and one patient (4.5%) had visceral metastasis.

PSMA PET/CT had a higher detection rate of extra-osseous lesions than CT alone (*p* = 0.023). Six patients (27.3%) had extra-osseous lesions identified by both PSMA PET/CT and CT alone, seven patients (31.8%) had lesions identified only by PSMA PET/CT, and nine patients (40.9%) had no lesions identified by either (Supplemental Table 5).

There was no significant difference in inter-reader agreement rates between PET/CT and CT alone (73% versus 68%, *p* = 1.0) (Supplemental Tables 3–4). Kappa coefficients for inter-reader comparisons were 0.44–0.72 for PSMA PET/CT and 0.46–0.59 for CT alone (Supplemental Table 6).

## Discussion

In this head-to-head, single-arm prospective trial of patients with prostate cancer who experienced biochemical progression during ADT, patients underwent both ^68^Ga-PSMA-11 PET/CT and ^99m^Tc-MDP planar bone scans, which were each interpreted by three blinded independent central readers. We report that PSMA PET/CT and planar bone scan plus CT had identical detection rates for bone metastasis. We also report that there was no significant difference in the number of detected bone metastases or inter-reader agreement. Finally, PSA level was not associated with higher detection rates of bone metastasis.

This study supports the accuracy and continued relevance of ^99m^Tc-MDP planar bone scans plus CT in the setting of biochemical progression during ADT. PSMA PET/CT scans are associated with increased health care costs and facilities equipped to deliver and interpret these scans are not accessible to patients in all communities^11^. Therefore, bone scan plus CT can serve as a cost-effective and accessible restaging modality for these patients without compromising the detection rates of bone metastasis.

Though this trial did not identify a difference in the detection rate of bone metastasis, there were significantly more extra-osseous metastases detected by PSMA PET/CT compared to CT alone. Overall, 7/22 patients (32%) in this cohort had extra-osseous metastasis detected only on PSMA PET/CT that were not visible on CT alone. There was no statistically significant difference in interreader agreement between PSMA PET/CT and CT alone.

These findings are broadly consistent with two other retrospective series of prostate cancer patients who progressed biochemically during ADT. A retrospective study of 200 patients by Fendler et al. reported M1 disease detected only on PSMA PET/CT in 55% of patients (vs. 32% in our study) with high inter-reader agreement (kappa, 0.81–0.91)^3^. A retrospective study of 55 patients by Weber et al. reported lesions detected only on PSMA PET/CT in 42% of patients with higher inter-reader agreement than CT alone (kappa, 0.77 vs. 0.29)^4^.

There were several limitations in this trial. First, this trial was closed prematurely. Second, there was heterogeneity in the patient population with regards to PSA level, prior treatment history, and current therapies. It is not known how these factors affect the detection rates of these two imaging modalities. Third, to reflect the clinical practice in the U.S., planar bone scans were used instead of single-photon emission computed tomography (SPECT)/CT. Many institutions outside of the U.S. are systematically using SPECT/CT to increase the sensitivity and accuracy of bone scans^12^. Use of SPECT/CT may have resulted in different results. Finally, there is a risk of false-positive findings on both PSMA PET/CT and bone scan^1,13^. It is possible that lesions detected only on one modality but not the other may not represent real disease. This trial did not include long-term clinical or radiographic follow-up of detected lesions or recommend biopsies to confirm the presence of active disease. Further studies are warranted.

In conclusion, ^68^Ga-PSMA-11 PET/CT and ^99m^Tc-MDP planar bone scan plus CT had identical bone metastasis detection rates with no significant difference in the number of detected bone lesions. In situations where cost and accessibility may limit access to PSMA PET/CT scans, bone scan plus CT can continue to serve as a cost-effective and accessible restaging modality in patients with biochemical progression during ADT.

## Supplementary Information

Supplementary Material 1.

Supplementary Material 2.

Supplementary Material 3.

Supplementary Material 4.

## Data Availability

The data is stored on an institutional repository and is available upon request to the corresponding author.
