# Supplementary material for: Phase 2 trial of PSMA PET CT versus planar bone scan and CT in prostate cancer patients progressing while on androgen deprivation therapy
Source: Sci Rep. 2024 Oct 18;14:24411. doi: 10.1038/s41598-024-75589-6 (PMC11487247; doi:10.1038/s41598-024-75589-6)
Supplement: Supplementary file 1 — Supplementary Material 1. [file 41598_2024_75589_MOESM1_ESM.pdf]

Case Report Form for PSMA PET/CT Readers

1. ≥1 positive bone lesions (M1b) on PSMA-PET?
   - No
   - Yes
     a. If positive bone disease, how many bone tumor lesions were detected on PSMA-PET?
        - 1
        - 2
        - 3
        - 4
        - 5
        - 6-20
        - >=20
        - Diffuse

   b. If positive bone disease, provide location (y/n)
      - In the Spine:
      - In the Pelvis:
      - In the Extremities:
      - In the Skull:
      - In the ribs/Sternum/Scapula/Claviculae:

2. ≥1 sclerotic bone lesions (M1b) on CT?
   - No
   - Yes

   a. If sclerotic bone disease on CT, how many bone tumor lesions were detected on CT?
      - 1
      - 2
      - 3
      - 4
      - 5
      - 6-20
      - >=20
      - Diffuse

If positive bone disease, provide location (y/n)
- In the Spine:
- In the Pelvis:
- In the Extremities:
- In the Skull:
- In the ribs/Sternum/Scapula/Claviculae:

3. Presence of PSMA negative bone lytic Lesions of ≥10 mm on CT?
   if yes, please provide localization and number:

4. ≥1 positive local lesion (T, prostate fossa) on PSMA-PET?
   - No
   - Yes.
     a. Is the tumor lesion positive on CT (≥10 mm)?

5. ≥1 positive pelvic lymph nodes (N1) on PSMA-PET?
   - No
   - Yes.
     b. If yes, Is the region positive on (≥15 mm)?

Presence of PSMA negative LN Lesions of ≥15 mm on CT?

6. ≥1 positive distant lymph nodes (M1a) on PSMA-PET?
   - No
   - Yes.
     a. If yes, provide location (y/n)
        Inguinal :
            Is the region positive on CT (≥15 mm)?
        Abdominal :
            Is the region positive on CT (≥15 mm)?
        Thoracic :
            Is the region positive on CT (≥15 mm)?
        Axillary :
            Is the region positive on CT (≥15 mm)?
        SupraClavicular :
            Is the region positive on CT (≥15 mm)?

Presence of PSMA negative LN Lesions of ≥15 mm on CT?

7. ≥1 positive visceral metastases (M1c) on PSMA-PET?
   - No
   - Yes.
     a. If yes, provide location (y/n)
        Lung
            Is the region positive on CT (≥10 mm)?
        Liver
            Is the region positive on CT (≥10 mm)?
        Brain
            Is the region positive on CT (≥10 mm)?
        Penile
            Is the region positive on CT (≥10 mm)?
        Other:___
            Is the region positive on CT (≥10 mm)?

Presence of PSMA negative soft tissue Lesions of ≥10 mm on CT?
