# Supplementary material for: Phase 2 trial of PSMA PET CT versus planar bone scan and CT in prostate cancer patients progressing while on androgen deprivation therapy
Source: Sci Rep. 2024 Oct 18;14:24411. doi: 10.1038/s41598-024-75589-6 (PMC11487247; doi:10.1038/s41598-024-75589-6)
Supplement: Supplementary file 2 — Supplementary Material 2. [file 41598_2024_75589_MOESM2_ESM.pdf]

Case Report Form for Bone Scan Readers

1. ≥1 positive bone lesions on bone scan?
    - No
    - Yes

2. If positive bone disease, how many bone tumor lesions were detected on bone scan?
    - 1
    - 2
    - 3
    - 4
    - 5
    - 6-20
    - >=20
    - Diffuse

If positive bone disease, provide location (y/n)
- In the Spine:
- In the Pelvis:
- In the Extremities:
- In the Skull:
- In the ribs/Sternum/Scapula/Claviculae:
