# Supplementary material for: Phase 2 trial of PSMA PET CT versus planar bone scan and CT in prostate cancer patients progressing while on androgen deprivation therapy
Source: Sci Rep. 2024 Oct 18;14:24411. doi: 10.1038/s41598-024-75589-6 (PMC11487247; doi:10.1038/s41598-024-75589-6)
Supplement: Supplementary file 3 — Supplementary Material 3. [file 41598_2024_75589_MOESM3_ESM.pdf]

Head-to-head comparison of ^{68}Ga-PSMA-11 PET/CT with ^{99m}Tc-MDP bone scan and CT for detection of M1b disease in prostate cancer patients with biochemical progression during ADT

Protocol Number: 21-000102

Protocol short title: PSMA vs BS/CT in CRPC

Protocol acronym: PSMAvsBS

National Clinical Trial (NCT) Identified Number: TBD

UCLA IRB # 21-000102

Principal Investigator: Jeremie Calais
Co-Principal Investigator: Johannes Czernin, Matthias Benz, Andrei Gafita

Ahmanson Translational Theranostics Division,
Department of Molecular and Medical Pharmacology.
David Geffen school of Medicine at UCLA

Version Number: v.1.0, 1/13/2021, rev 2/9/2021, 5/21/2021, 7/22/21

Summary of Changes from Previous Version:

**Affected Section(s)**	**Amendment, Version, and Date**	**Summary of Revisions Made**	**Rationale**
1.2, 2.2, 2.3.2, 6.1.2, 6.2.3, 10.1.7	A1, V2, 07/22/2021	- Final report within 5 business days<br>- Include FDA label from IND<br>- Remove "no fasting is required" and update imaging protocol to scan upper-thigh	- Report requires more time to be finalized<br>- PSMA scan approved by FDA<br>- Fasting is required

Table of Contents

STATEMENT OF COMPLIANCE 4
1 PROTOCOL SUMMARY 5
1.1 Synopsis 5
1.2 Schema 7
1.3 Schedule of Activities (SoA) 8
2 INTRODUCTION 8
2.1 Study Rationale 8
2.2 Background 8
2.3 Risk/Benefit Assessment 9
2.3.1 Known Potential Risks 10
2.3.2 Known Potential Benefits 10
2.3.3 Assessment of Potential Risks and Benefits 10
3 OBJECTIVES AND ENDPOINTS 11
4 STUDY DESIGN 11
4.1 Overall Design 11
4.2 Scientific Rationale for Study Design 11
4.3 End of Study Definition 12
5 STUDY POPULATION 12
5.1 Inclusion Criteria 12
5.2 Exclusion Criteria 12
5.3 Screen Failures 12
5.4 Strategies for Recruitment and Retention 13
6 STUDY INTERVENTION 13
6.1 Study Intervention(s) Administration 13
6.1.1 Study Intervention Description 13
6.1.2 Dosing and Administration 13
6.2 Preparation/Handling/Storage/Accountability 14
6.2.1 Acquisition and accountability 14
6.2.2 Formulation, Appearance, Packaging, and Labeling 14
6.2.3 Product Storage and Stability 15
7 STUDY INTERVENTION DISCONTINUATION AND PARTICIPANT DISCONTINUATION/WITHDRAWAL 15
7.1 Discontinuation of Study Intervention 15
7.2 Participant Discontinuation/Withdrawal from the Study 15
7.3 Lost to Follow-Up 16
8 STUDY ASSESSMENTS AND PROCEDURES 16
8.1 Efficacy Assessments 16
8.2 Safety and Other Assessments 17
8.3 Adverse Events and Serious Adverse Events 17
8.3.1 Definition of Adverse Events (AE) 17

8.3.2 Definition of Serious Adverse Events (SAE) 17
8.3.3 Classification of an Adverse Event 18
8.3.4 Time Period and Frequency for Event Assessment and Follow-Up 19
8.3.5 Adverse Event Reporting 20
8.3.6 Serious Adverse Event Reporting 21
9 STATISTICAL CONSIDERATIONS 21
9.1 Statistical Hypotheses 21
9.2 Sample Size Determination 22
9.3 Populations for Analyses .... **Error! Bookmark not defined.**
9.4 Statistical Analyses 22
9.4.1 General Approach .... **Error! Bookmark not defined.**
9.4.2 Analysis of the Primary Efficacy Endpoint(s) .... **Error! Bookmark not defined.**
9.4.3 Analysis of the Secondary Endpoint(s) . **Error! Bookmark not defined.**
9.4.4 Safety Analyses .... **Error! Bookmark not defined.**
9.4.5 Baseline Descriptive Statistics .... **Error! Bookmark not defined.**
9.4.6 Planned Interim Analyses .... **Error! Bookmark not defined.**
9.4.7 Sub-Group Analyses .... **Error! Bookmark not defined.**
9.4.8 Tabulation of Individual participant Data .... **Error! Bookmark not defined.**
9.4.9 Exploratory Analyses .... **Error! Bookmark not defined.**
10 SUPPORTING DOCUMENTATION AND OPERATIONAL CONSIDERATIONS 24
10.1 Regulatory, Ethical, and Study Oversight Considerations 24
10.1.1 Informed Consent Process 24
10.1.2 Study Discontinuation and Closure 24
10.1.3 Confidentiality and Privacy 24
10.1.4 Future Use of Stored Specimens and Data 25
10.1.5 Key Roles and Study Governance 25
10.1.6 Safety Oversight 26
10.1.7 Clinical Monitoring 26
10.1.8 Quality Assurance and Quality Control 26
10.1.9 Data Handling and Record Keeping 26
10.1.10 Protocol Deviations 27
10.1.11 Publication and Data Sharing Policy 27
10.1.12 Conflict of Interest Policy 27
10.2 Additional Considerations 27
10.3 Abbreviations 28
10.4 Protocol Amendment History 28
11 References 28

STATEMENT OF COMPLIANCE

The trial will be conducted in accordance with International Conference on Harmonisation Good Clinical Practice (ICH GCP), and applicable United States (US) Code of Federal Regulations (CFR). The Principal Investigator will assure that no deviation from, or changes to the protocol will take place without prior agreement from the Institutional Review Board (IRB), except where necessary to eliminate an immediate hazard(s) to the trial participants. All personnel involved in the conduct of this study have completed Human Subjects Protection and ICH GCP Training.

The protocol, informed consent form(s), recruitment materials, and all participant materials will be submitted to UCLA IRB for review and approval. Approval of both the protocol and the consent form must be obtained before any participant is enrolled. Any amendment to the protocol will require review and approval by the IRB before the changes are implemented in the study. All changes to the consent form will be IRB approved; a determination will be made regarding whether a new consent needs to be obtained from participants who provided consent, using a previously approved consent form.

PROTOCOL SUMMARY

SYNOPSIS

**Title:**	Head-to-head comparison of ^{68}Ga-PSMA-11 PET/CT with ^{99m}Tc-MDP bone scan and CT for detection of M1b disease in prostate cancer patients with biochemical progression during ADT.
**Study Description:**	This is a prospective, single-center, single-arm, open-label, head-to-head comparison imaging study in 102 prostate cancer (PCa) patients progressing during androgen deprivation therapy (ADT). Patients are eligible for enrollment irrespective of whether the patient has preexisting non-metastatic or metastatic, castration sensitive or castration resistant disease. The aim of the study is to compare the detection rate of ^{68}Ga-PSMA-11 PET/CT versus ^{99m}Tc-MDP bone scan and CT (BS/CT) for M1b disease (bone metastases).<br><br>^{68}Ga-PSMA PET/CT will be compared to ^{99m}Tc-MDP bone scan and the CT component of the PET/CT. Therefore, no additional CT needs to be acquired. Each imaging modality will be read by three blinded independent central readers (BICR).
**Primary Objective:**	To compare the per-patient detection rate of ^{68}Ga-PSMA-11 PET/CT versus BS/CT for M1b in patients treated with androgen deprivation therapy (ADT) who are referred for evaluation of disease progression.
**Secondary Objective:**<br><br><br><br><br><br><br>**Sub-group Analysis:**	1. To compare the number of lesions rated as positive for bone metastases between the two imaging tests.<br>2. To compare the detection rate of ^{68}Ga-PSMA PET/CT versus BS/CT for all M1 disease (M1a or M1b or M1c).<br>3. To compare the positive predictive value (PPV) per-patient in patients with available lesion validation (follow up imaging or biopsy) that may be performed as standard of care based on the treating physician's decision).<br><br>Subset analysis based on PSADT (≤ 6 month vs. > 6 months)
**Study Population:**	Sample size: 102<br><br>*Inclusion criteria:*

	1. Patient with biochemical progression during ADT or combination therapies including ADT who are referred for imaging evaluation (PSA level ≥1 ng/ml that has increased on at least 2 successive occasions at least 1 weak apart).<br>2. Patients with bone scan scheduled or performed<br>a. within 30 days of the PSMA PET, without any new PCa therapy in between<br>b. bone scans performed at UCLA and external institutions are eligible if DICOM images can be obtained, imported, and anonymized<br>3. Patients enrolled in other clinical trials are eligible if they satisfy all other criteria of eligibility<br>4. Patients or their legal representatives must have the ability to read, understand and provide written informed consent<br><br>Exclusion criteria:<br>1. Initiation of a new therapy between the PSMA PET/CT and the bone scans<br>2. Inability to provide written informed consent<br>3. Known inability to remain still and lie flat for duration of each imaging procedure (about 30 minutes)
**Study Phase/Design:**	Single-center, open-label, single-arm, head-to-head comparison, prospective phase 2 study.
**Description of Sites/Facilities Enrolling Participants:**	All PSMA PET/CT will be conducted at UCLA nuclear medicine.<br>^{68}Ga-PSMA-11 will be prepared in the UCLA Biomedical Cyclotron facility.<br>^{99m}Tc-MDP bone scans performed as per-standard of care (SOC) at UCLA or outside of UCLA will be used for the analysis.<br>Patients will be referred by their treating urologist, uro-oncologist, uro-oncologist, or radiation oncologist.
**Description of Study Intervention:**	- 1 PSMA PET scan: 3-7 mCi intravenous bolus injection of ^{68}Ga-PSMA-11
**Study Duration:**	Estimated study duration from enrollment until completion of data analyses is 24 months.
**Participant Duration:**	Participant duration can be up to 1 month (time interval between the 2 scans). Follow-up will be performed indirectly via the referring uro-oncologist, therefore patients will not undergo any follow-up procedure as part of the study.

1.2 SCHEMA

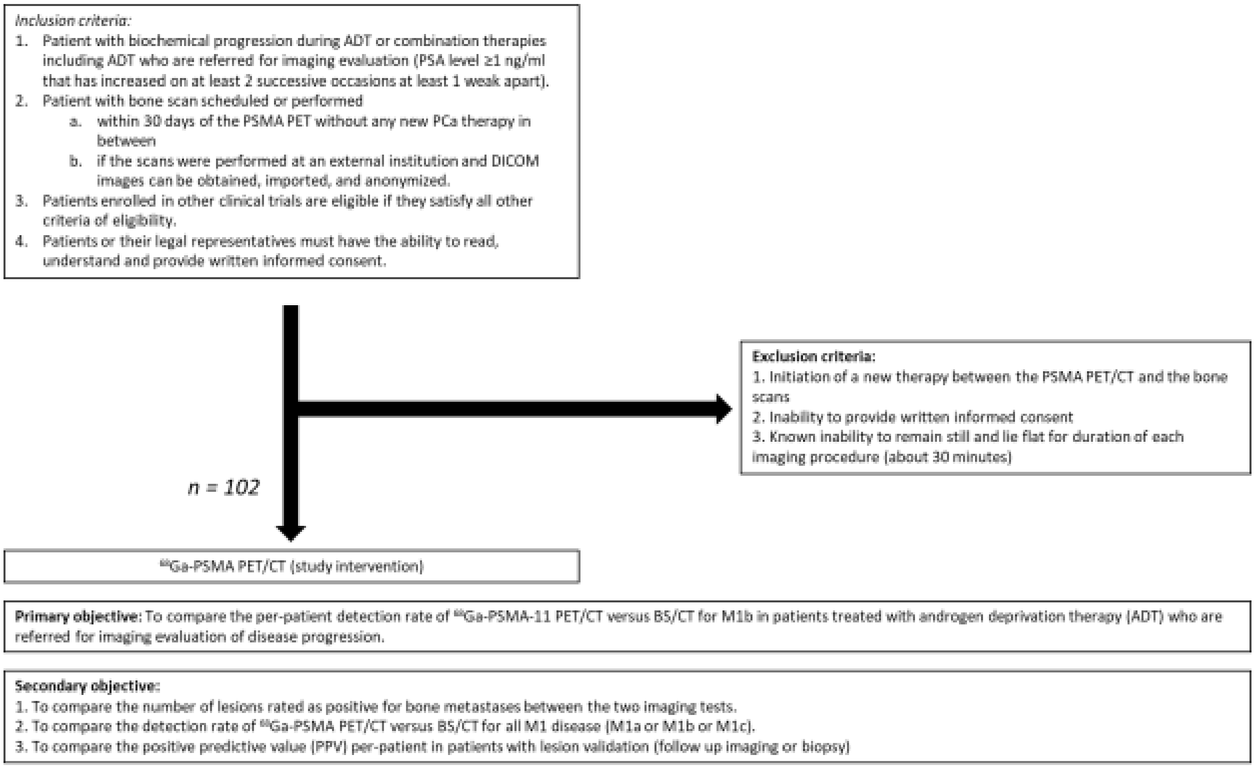
Inclusion criteria:
1. Patient with biochemical progression during ADT or combination therapies including ADT who are referred for imaging evaluation (PSA level ≥1 ng/ml that has increased on at least 2 successive occasions at least 1 weak apart).
2. Patient with bone scan scheduled or performed
a. within 30 days of the PSMA PET without any new PCa therapy in between
b. if the scans were performed at an external institution and DICOM images can be obtained, imported, and anonymized.
3. Patients enrolled in other clinical trials are eligible if they satisfy all other criteria of eligibility.
4. Patients or their legal representatives must have the ability to read, understand and provide written informed consent.
Exclusion criteria:
1. Initiation of a new therapy between the PSMA PET/CT and the bone scans
2. Inability to provide written informed consent
3. Known inability to remain still and lie flat for duration of each imaging procedure (about 30 minutes)
n = 102
^{68}Ga-PSMA PET/CT (study intervention)
Primary objective: To compare the per-patient detection rate of ^{68}Ga-PSMA-11 PET/CT versus BS/CT for M1b in patients treated with androgen deprivation therapy (ADT) who are referred for imaging evaluation of disease progression.
Secondary objective:
1. To compare the number of lesions rated as positive for bone metastases between the two imaging tests.
2. To compare the detection rate of ^{68}Ga-PSMA PET/CT versus BS/CT for all M1 disease (M1a or M1b or M1c).
3. To compare the positive predictive value (PPV) per-patient in patients with lesion validation (follow up imaging or biopsy)

SCHEDULE OF ACTIVITIES (SOA)

**Procedures**	Pre-screening	Clinic visit day	Within 5 business days of clinic visit	Within 1 month of clinic visit
Obtaining DICOM bone scan				X
Pre-screening	X			
Demographics	X			
Medical history	X			
Informed consent		X		
Administer ^{68}Ga-PSMA-11		X		
PET/CT scan		X		
Adverse event monitoring		X		
Complete Case Report Forms (CRFs)		X		
Final imaging report			X	

INTRODUCTION

STUDY RATIONALE

Literature on the detection rate and on patient impact of PSMA PET/CT in comparison to conventional imaging in patients treated with androgen deprivation therapy (ADT) who are referred for imaging evaluation of disease progression is limited. Major limitations of these studies include their retrospective nature and inclusion of patients with negative findings on conventional imaging.

The aim of the present study is to investigate the detection rate of M1b of ^{68}Ga-PSMA PET/CT in comparison to conventional imaging in patients treated with ADT or combination treatments including ADT who are referred for imaging evaluation of disease progression.

BACKGROUND

Prostate cancer continues to be the leading cancer diagnosis in males. In 2020, it is estimated that there will be 191,930 new cases of prostate cancer (10.6% of all new cancer cases). In men, prostate cancer has the second highest mortality rate (after lung cancer) with 33.330 estimated deaths (5.5% of all cancer deaths).

On Dec 1, 2020, FDA approved first PSMA-targeted PET imaging drug for men with prostate cancer: ^{68}Ga-PSMA-11(https://www.fda.gov/news-events/press-announcements/fda-approves-first-psma-targeted-pet-imaging-drug-men-prostate-cancer). Ga 68 PSMA-11 is indicated for patients with suspected prostate cancer metastasis. It is

also indicated for patients with suspected prostate cancer recurrence based on elevated serum prostate-specific antigen (PSA) levels.

In a multicenter, two-arm, randomized clinical study, PSMA PET/CT has shown superior accuracy in comparison to conventional imaging for the initial staging of high-risk prostate cancer patients (proPSMA)[1]. The accuracy was 92% and 65% for PSMA PET/CT and conventional imaging, respectively. In a recent prospective, multicenter trial designed for regulatory approval, PSMA-PET/CT demonstrated high detection rate and positive predictive value for the localization of recurrent prostate cancer [2].

The added value of PSMA PET in comparison to conventional imaging, especially for detecting bone involvement, in patients treated with ADT or combination treatments including ADT who are referred for imaging evaluation of disease progression is less well studied [3, 4].

The American (National Comprehensive Cancer Network; NCCN) and European (European Association of Urology; EAU) guidelines for the management of prostate cancer suggest "conventional imaging" for the assessment of imaging progression in patients treated with ADT. Conventional imaging consists of a ^{99m}Tc-MDP bone scan (bone scan) for the evaluation of bone metastases and computed tomography (CT) for the assessment of lymph node and visceral metastases. However, conventional imaging has shown insufficient sensitivity and specificity to detect non-localized disease. In the setting of non-metastatic castration resistant prostate cancer progressing on ADT, which corresponds to the study population investigated in this proposed study, bone scans are rarely positive in asymptomatic men with a PSA < 10ng/ml [5]. Since relative risk for bone metastasis or death increases as the PSA doubling time (PSAdt) shortens, bone scan is recommended to be performed when the PSAdt is ≤ 8 months. In addition, CT is known to underestimate the lymph node involvement in prostate cancer compared with PSMA-PET [3].

Retrospective studies have shown the superiority of PSMA-PET in metastatic castration-resistant prostate cancer in comparison to conventional imaging even at low PSA levels. Fendler et al. [3] retrospectively analyzed 200 patients with non-metastatic castration-resistant prostate cancer (nmCRPC) who were negative on conventional imaging and subsequently received a PSMA PET/CT. Patients had a prostate-specific antigen (PSA) >2 ng/mL and were at high risk for metastatic disease (PSAdt of ≤10 months and/or Gleason score of ≥8). Despite negative conventional imaging, PSMA-PET was positive in 196 of 200 patients. Overall, 44% had pelvic disease, including 24% with local prostate bed recurrence, and 55% had M1 disease despite negative conventional imaging. M1 disease was located in extrapelvic nodes, bone, and viscera in 39%, 24%, and 6% patients, respectively. Weber et al [4] assessed the value of PSMA-PET/CT in the detection of early CRPC with a PSA ≤3 ng/mL. PSMA-PET/CT was positive in 41/55 (75%) patients. 16/55 (29%) patients had local disease only, 25/55 (45%) had M1-disease.

The treatment landscape of prostate cancer has changed substantially over the last couple of years with second line ADT and chemotherapy introduced earlier in the patient's prostate cancer disease continuum. Therefore, accurate upstaging using PSMA-PET will impact patient management in patients who progress under ADT or combination treatments including ADT.

RISK/BENEFIT ASSESSMENT

KNOWN POTENTIAL RISKS

The risk level of the ^{68}Ga-PSMA-11 imaging scan is low. The substance amount is so small that it does not affect the normal processes of the body. This research study involves exposure to a small amount of radiation from one ^{68}Ga-PSMA-11 PET scan.

^{68}Ga-PSMA-11 is a microdose radiopharmaceutical which does not cause any systematic effect on human body. Of 960 patients from two prospective bi-center studies (submitted to FDA in NDA 212642 and NDA 212643), there were no deaths, SAEs, other significant AEs, or discontinuations due to AEs, and a total of 26 AEs were reported, all of which are classified as CTCAE Grade 1. Among these 26 AEs, 73.1% were classified as unrelated or unlikely related to the study drug, and the rest were classified as possibly or probably related to the study drug. The most common reported AEs were paresthesia and diarrhea which occurred in 2 patients each.

The typical and recommended activity of ^{68}Ga-PSMA-11 Injection (5mCi, 185 MBq) resulted in a whole-body absorbed dose of 3.12 mSv, which is comparable to effective radiation dose of 3.15 mSv/150MBq for ^{68}Ga-DOTATATE (NETSPOT®), and 8 mSv/370 MBq for ^{18}F-fluciclovine (Axumin®). ^{68}Ga-PSMA-11 injection, similar to other radiopharmaceuticals, contributes minimally to a patient's overall long-term cumulative radiation exposure.

^{99m}Tc-MDP bone scan and the CT is a standard of care procedure and therefore not part of the radiation risk assessment. The effective dose will be below the annual dose limit of 50 mSv from cumulative studies as outlined in the FDA guidance for RDRC human research [6].

KNOWN POTENTIAL BENEFITS

This study is not designed for participant's individual benefit.

As approved in NDA 212642 on Dec 1, 2020, Ga 68 PSMA-11 Injection is a radioactive diagnostic agent indicated for positron emission tomography (PET) of prostate-specific membrane antigen (PSMA) positive lesions in men with prostate cancer:

• with suspected metastasis who are candidates for initial definitive therapy.

• with suspected recurrence based on elevated serum prostate-specific antigen (PSA) level

It is yet unknown whether PSMA PET/CT can improve the detection of bone metastases.

ASSESSMENT OF POTENTIAL RISKS AND BENEFITS

This study is not designed for participant's individual benefit but based on previous studies (see section 2.3.1) the risk of ^{68}Ga-PSMA-11 PET/CT is very low. However, PCa patients may later benefit from the knowledge acquired in this study.

OBJECTIVES AND ENDPOINTS

Primary Objective:

- To compare the per-patient detection rate of ^{68}Ga-PSMA-11 PET/CT versus BS/CT for M1b in patients treated with androgen deprivation therapy (ADT) who are referred for the imaging evaluation of disease progression.

Secondary Objectives:

- To compare the number of lesions rated as positive for bone metastases between the two imaging tests.
- To compare the detection rate of ^{68}Ga-PSMA PET/CT versus BS/CT for all M1 disease (M1a or M1b or M1c).
- To compare the positive predictive value (PPV) per-patient in patients with available lesion validation (follow-up imaging or biopsy that may be performed as standard of care based on the treating physician's decision).

Sub-set Analysis:

- Subset analysis based on PSAdt (≤ 6 month vs. > 6 months)

STUDY DESIGN

OVERALL DESIGN

This is a prospective, single-center, single-arm, open-label, head-to-head comparison imaging study in 102 patients with biochemical progression under ADT or combination treatments including ADT and who are referred for imaging evaluation of disease progression. Patients are eligible for enrollment irrespective of whether the patient has preexisting non-metastatic or metastatic, castration sensitive or castration resistant disease. The key objective of the study is to compare the detection rate of ^{68}Ga-PSMA-11 PET/CT versus ^{99m}Tc-MDP bone scan and CT for M1b disease.

Eligible participants will undergo ^{68}Ga-PSMA-11 PET/CT (study procedure) and ^{99m}Tc-MDP bone scan (standard of care) within 1 month. Three blinded independent central readers (BICR) will read each scan.

The ^{68}Ga-PSMA-11 PET/CT will be compared to the ^{99m}Tc-MDP bone scan and the CT component of the PET/CT. Therefore, no additional CT needs to be acquired.

SCIENTIFIC RATIONALE FOR STUDY DESIGN

The treatment landscape of prostate cancer has changed substantially over the last couple of years with second line ADT and chemotherapy introduced earlier in the patient's prostate cancer disease continuum. Conventional imaging (CT and bone scan) is known to have limited sensitivity in comparison to PSMA PET at low PSA values in patients who progress under ADT. Therefore, accurate upstaging using PSMA-PET will impact patient management in these patients. Patients with negative conventional imaging but M1b disease as per ^{68}Ga-PSMA PET/CT will migrate e.g. from nmCRPC to mCRPC.

In cases of single- or oligometastatic disease on conventional imaging in which PSMA PET will detect additional lesions, patients will migrate to polymetastatic disease. This will enable earlier access to systemic treatments such as taxane-based chemotherapy or next-generation hormones (Abiraterone / Enzalutamide).

END OF STUDY DEFINITION

A participant is considered to have completed the study if he has completed the bone scan and ^{68}Ga-PSMA-11 PET/CT, including the procedures shown in the Schedule of Activities (SoA), Section 1.3.
The end of the study is defined as completion of procedures shown in the SoA.

STUDY POPULATION

INCLUSION CRITERIA

In order to be eligible to participate in this study, an individual must meet the following criteria:

1. Patient with biochemical progression during ADT or combination therapies including ADT who are referred for imaging evaluation (PSA level ≥1 ng/ml that has increased on at least 2 successive occasions at least 1 weak apart).
2. Patients with bone scan scheduled or performed
    a. within 30 days of the PSMA PET, without any new PCa therapy in between
    b. bone scans performed at UCLA and external institutions are eligible if DICOM images can be obtained, imported, and anonymized
3. Patients enrolled in other clinical trials are eligible if they satisfy all other criteria of eligibility
4. Patients or their legal representatives must have the ability to read, understand and provide written informed consent

EXCLUSION CRITERIA

Individual who meets any of the following criteria will be excluded from participation in this study:

1. Initiation of a new therapy between the PSMA PET/CT and the bone scans
2. Inability to provide written informed consent
3. Known inability to remain still and lie flat for duration of each imaging procedure (about 30 minutes)

SCREEN FAILURES

Screen failures are defined as participants who consent to participate in the clinical trial but are not subsequently entered in the study.

STRATEGIES FOR RECRUITMENT AND RETENTION

Patients will be informed about the study by their treating urologist, uro-oncologist or radiation oncologist. Treating physicians will refer the potentially eligible volunteer patients to the UCLA Nuclear Medicine research team by providing the required PCa history, medical records, and inclusion and exclusion criteria.
UCLA Nuclear Medicine will pre-screen via phone and assess the eligibility of the patient based on the medical records and documents provided by the referring physician.
Flyers for referring physicians and clinicaltrials.gov registration will be utilized for patient recruitment information.
Participants will not be compensated or provided any incentives for the study participation. The study participant will not be responsible for the payment of the PSMA PET scans. Patients will be fully responsible for the payment of the whole body bone scans as these are standard of care procedures and eligible for insurance coverage.

STUDY INTERVENTION: 68GA-PSMA-11 PET/CT

STUDY INTERVENTION(S) ADMINISTRATION

STUDY INTERVENTION DESCRIPTION

A single dose activity of ^{68}Ga-PSMA-11 will be intravenously administered per PET scan to the patient as a bolus injection. After 50-100 minutes of uptake time, the patient will undergo a whole body (skull to mid-thighs) PET/CT study.

DOSING AND ADMINISTRATION

5 mCi of ^{68}Ga-PSMA-11 dose (range of 3-7 mCi) will be administered intravenously as a bolus injection for each PSMA PET scan. The patient will stay in the clinic to allow 50-100 mins radiotracer uptake. A whole-body PET/CT study will be acquired afterwards.

Imaging protocol:
Oral hydration is recommended immediately after injection of the radiotracer, before start of the scan. ^{68}Ga-PSMA-11 PET preparation and injection:

1. PET/CT images will be obtained using the Siemens Biograph 64 and mCT scanners.
2. PET/CT images will be acquired at 50-100 minutes after intravenous injection of 111-259 MBq (3-7 mCi) of ^{68}Ga-PSMA-11.
3. PET/CT scan coverage will extend from upper-thigh to the vertex.
4. A diagnostic CT will be acquired just before the PET imaging acquisition for attenuation correction.

5. CT-Contrast may be administered if requested by the referring clinician or the attending nuclear medicine physician. Details are outside the scope of this study protocol.

6. PET images will be acquired applying a weight-based protocol (<170lbs: 2 min/bed; 170-200lbs: 3 min/bed; >200lbs: 4 min/bed). The total image acquisition time will be 20-50 min dependent on the subject's body weight and height (63).

7. The PET emission scan will be corrected for decay, dead-time, random events, and scatter. PET images will be corrected for attenuation using segmented attenuation data of the low-dose CT scan. PET images will be reconstructed using ordered subset expectation maximization (OSEM) and filtered to a spatial resolution of 5 mm (isotropic) with a Gauss-filter.

8. DICOM images will be anonymized.

9. Clinical reports will be used to guide follow-up.

PREPARATION/HANDLING/STORAGE/ACCOUNTABILITY

ACQUISITION AND ACCOUNTABILITY

^{68}Ga-PSMA-11 will be provided by UCLA Biomedical Cyclotron. Upon receiving the drug product, a certified Nuclear Medicine technologist will perform dose calibration and fill the electronic radiopharmacy log. Any unused drug products will be disposed of according to local radiation safety guidelines.

FORMULATION, APPEARANCE, PACKAGING, AND LABELING

PSMA-11 Ga 68 injection is a positron emitting radiopharmaceutical containing radioactive 68Ga-labeled Glu-NH-CO-NH-Lys(Ahx)-HBED-CC (PSMA-11), which is used for diagnostic purposes in conjunction with Positron Emission Tomography (PET). PSMA-11 Ga 68 Injection is a sterile, pyrogen free, clear, colorless aqueous solution presented in a multi-dose vial.

The final drug product volume is 12 mL and contains 10 mL Sodium Chloride 0.9% Injection, 1 mL of ethanol and 1 mL of Water for Injection. The solution has a pH between 4.0 and 7.0 and each mL of PSMA-11 Ga 68 Injection contains between 0.5 – 5 mCi (18.5 – 185 MBq) 68Ga-PSMA-11 at the end of synthesis (EOS).

The product vial label is attached below.

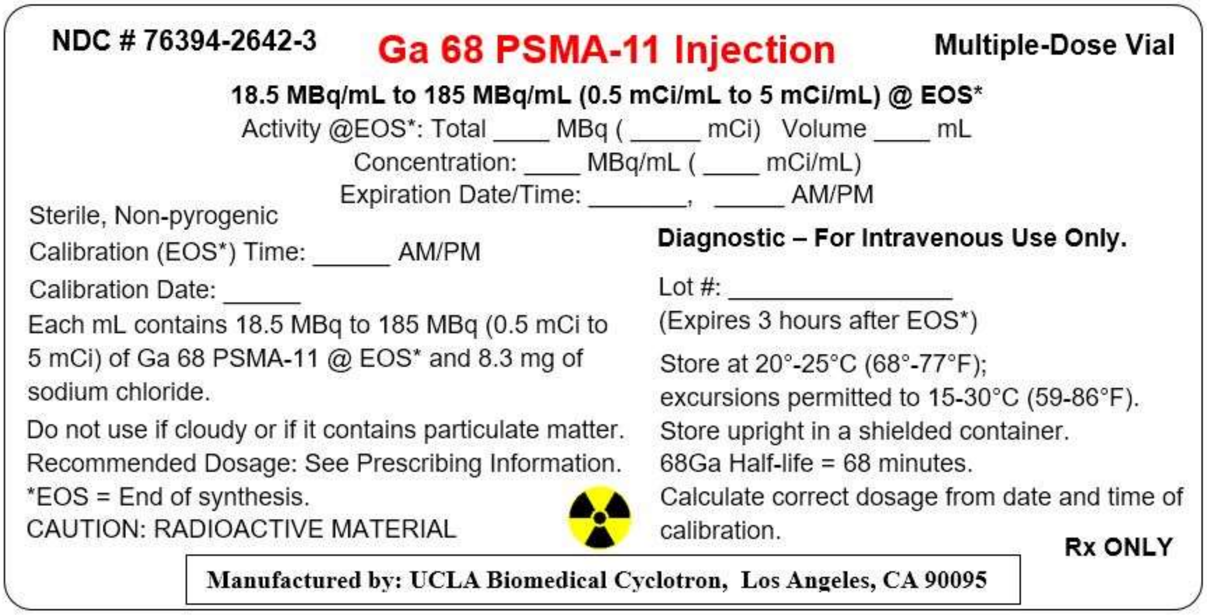

PRODUCT STORAGE AND STABILITY

Store Ga 68 PSMA-11 Injection upright in a lead shielded container at 25°C (77°F); excursions are permitted from 15°C to 30°C (59°F to 86°F). Store Ga 68 PSMA-11 Injection within the original container in radiation shielding. Use Ga 68 PSMA-11 Injection within 3 hours of end of synthesis time.

STUDY INTERVENTION DISCONTINUATION AND PARTICIPANT DISCONTINUATION/WITHDRAWAL

DISCONTINUATION OF STUDY INTERVENTION

The investigators may withdraw subjects from the study for one or more of the following reasons: failure to follow the instructions of the Principal Investigator and/or study staff; determination that continuing the participation could be harmful to the subject; the study is cancelled or other administrative reasons.

Any patient who does not complete the PSMA PET/CT scan and the bone scan will be excluded from the final analysis.

Any patient who receives a new therapy for prostate cancer between the PSMA PET/CT scan and the bone scan will be excluded from the final analysis.

PARTICIPANT DISCONTINUATION/WITHDRAWAL FROM THE STUDY

The patient has the right to withdraw from the study and discontinue participation at any time.

LOST TO FOLLOW-UP

A participant will be considered lost to follow-up if he fails to undergo the PSMA PET/CT and is unable to be contacted by the study site staff.

The following actions must be taken if a participant fails to appear in the clinic for the required study visit:

- The site will attempt to contact the participant and reschedule the missed visit within the time frame allowed by the SoA and ascertain if the participant wishes to and/or should continue in the study.
- Before a participant is deemed lost to follow-up, the investigator or designee will make every effort to regain contact with the participant (where possible, 3 telephone calls and, if necessary, a certified letter to the participant's last known mailing address or local equivalent methods). These contact attempts should be documented in the participant's study file.
- Should the participant continue to be unreachable, he will be considered to have withdrawn from the study with a primary reason of lost to follow-up.

STUDY ASSESSMENTS AND PROCEDURES

EFFICACY ASSESSMENTS

Image Analysis

Imaging data will be anonymized and collected at UCLA Nuclear Medicine.

PSMA-PET/CT images will be reviewed and analyzed by three blinded independent central readers (BICR).

BICR will be provided with anonymized DICOM datasets, a reading collection form and reading guidelines.

For PSMA PET/CT, readers will be asked to follow the miTNM PROMISE criteria [7]. Five anatomic regions of suspected disease (T, N, M1a, M1b, M1c) will be graded on a two-point scale by the reader (0=Negative or 1= Positive). The total number of PSMA-positive cancer lesions will be recorded for each region. The anatomic localization of each lesion will also be recorded. 3D volumes of interest (VOI) will be used to record maximum standardized uptake values (SUV_{max}) for each suspected cancer lesion.

For bone scan and CT, readers will be asked to grade as positive or negative the five anatomic regions of suspected disease (T, N, M1a, M1b, M1c). The total number of suspected cancer lesions will be recorded for each region. The anatomic localization of each lesion will also be recorded.

Follow-up for lesion validation

Local Interpretations: PSMA PET/CT and bone scan images will initially be interpreted by a board certified nuclear medicine physician and/or a board-certified radiologist at the time of the imaging study at the institution that the study

is being performed. These interpretations will not be used for final evaluation and will not be reported as part of the primary or secondary endpoints. The reports will be used by the local investigator to obtain follow-up of positive findings verification.

Further patient management and treatment strategy will be performed according to the treating physician's decision. Therefore, potential follow-up imaging and / or potential biopsy of equivocal imaging finding might be performed in a subset of patients as a standard of care procedure according to the treating physician's decision and are not part of this study. The investigator however will try to obtain the results of serum PSA, imaging follow-up or biopsy if performed.

A lesion will be considered as positive if:

- confirmed on another imaging modality (MRI, choline PET, fluciclovine PET),
- change of a bone lesion to sclerotic or blastic on follow-up CT imaging,
- showed increase (+25%) or decrease (-25%) in size or uptake in correlation by anatomic imaging / PSMA-PET with PSA response,
- confirmed as PCa by histopathology,
- PSA response (-25%) after focal metastasis-directed therapy to the lesion without any systemic therapy

SAFETY AND OTHER ASSESSMENTS

Participants will be monitored for any potential adverse events during clinic visit, and will be instructed to contact the research team to report any adverse experience.

ADVERSE EVENTS AND SERIOUS ADVERSE EVENTS

DEFINITION OF ADVERSE EVENTS (AE)

Adverse event means any untoward medical occurrence associated with the use of an intervention in humans, whether or not considered intervention-related (21 CFR 312.32 (a).

DEFINITION OF SERIOUS ADVERSE EVENTS (SAE)

An adverse event (AE) or suspected adverse reaction is considered "serious" if, in the view of either the investigator or sponsor, it results in any of the following outcomes: death, a life-threatening adverse event, inpatient hospitalization or prolongation of existing hospitalization, a persistent or significant incapacity or substantial disruption of the ability to conduct normal life functions, or a congenital anomaly/birth defect. Important medical events that may not result in death, be life-threatening, or require hospitalization may be considered serious when, based upon appropriate

medical judgment, they may jeopardize the participant and may require medical or surgical intervention to prevent one of the outcomes listed in this definition.

CLASSIFICATION OF AN ADVERSE EVENT

1.1.1.1 SEVERITY OF EVENT

CTCAE 4.03 will be used to assess the severity of adverse events.

1.1.1.2 RELATIONSHIP TO STUDY INTERVENTION

All grade 3 and above adverse events (AEs) must have their relationship to study intervention assessed by the clinician who examines and evaluates the participant based on temporal relationship and his/her clinical judgment. The degree of certainty about causality will be graded using the categories below. In a clinical trial, the study product must always be suspect.

**Relationship**	**Attribution**	**Description**
Unrelated to investigational drug/intervention	Unrelated	The AE *is clearly NOT related* to the intervention
	Unlikely	The AE *is doubtfully related* to the intervention
Related to investigational drug/intervention	Possible	The AE *may be related* to the intervention
	Probable	The AE *is likely related* to the intervention
	Definite	The AE *is clearly related* to the intervention

All adverse events (AEs) regardless of severity or relation to study participation, observed or reported by the participant during the protocol required AE collection period will be reported to the DSMB / IRB.

EXPECTEDNESS

Nuclear Medicine physician will be responsible for determining whether an adverse event (AE) is expected or unexpected. An AE will be considered unexpected if the nature, severity, or frequency of the event is not consistent with the risk information previously described for the study intervention.

Potential adverse events:

"Injection"
The administration of the PET tracer will feel like a slight pinprick as the tracer is given by intravenous injection.

"Side effects"
The substance amount is so small that it does not affect the normal processes of the body. This is why a PET probe is called a "tracer". Therefore, there is no pharmacologic side effect from the injection of the PET tracer. The subjects will not feel anything related to the radioactivity of the substance in their body.

"Confinement"

Patients who are claustrophobic may feel some anxiety while positioned in the scanner (5%). Also, some patients find it uncomfortable to hold one position for more than a few minutes.

"CT-Contrast"

Oral contrast

Mild and reversible side effects from oral CT contrast can include abdominal cramping, diarrhea, nausea, vomiting or constipation.

IV Contrast

IV Contrast injected through the IV can cause allergic reactions. It is estimated that the overall frequency of allergic reactions is below 1 percent. Most of these are very mild and may consist of only a few hives. However, in one of every 1,000 to 2,000 examinations, a moderate or severe reaction can occur. The risk of death from a contrast agent is estimated to be 0.3 to 2.6 per 100,000 uses

• Mild allergic reactions occur in below 1% of patients who receive intravenous contrast material. Allergic reactions to contrast may include itching, rash, hives, or a feeling of warmth throughout the body. These side effects usually disappear quickly. If needed, antihistamines can be given to help relieve the symptoms.

• Anaphylactic reaction is a severe allergic reaction. This is rare and occurs in only one of 1000 to 2000 patients. When this occurs, the patient may experience severe hives and/or difficulty in breathing. This reaction is quite rare, but is potentially life-threatening if not treated. Medications that may include corticosteroids, antihistamines, and epinephrine reverse this adverse reaction.

• Toxicity to kidneys: Toxicity to the kidneys that can result in kidney failure is an extremely rare complication of the intravenous contrast used in CT scans. Patients who already have impaired kidney function are most likely to get this reaction.

• Death due to allergic reactions is a very rare event. This occurs in 0.3 to 2.6 out of 100,000 patients in regards to the IV contrast for the CT portion (comparable in magnitude to the risk of death from receiving a dose of penicillin).

TIME PERIOD AND FREQUENCY FOR EVENT ASSESSMENT AND FOLLOW-UP

The occurrence of an adverse event (AE) or serious adverse event (SAE) may come to the attention of study personnel during study visits.

All AEs including local and systemic reactions not meeting the criteria for SAEs will be captured on the appropriate case report form (CRF). Information to be collected includes event description, time of onset, clinician's assessment of severity, relationship to study product (assessed only by those with the training and authority to make a diagnosis), and time of resolution/stabilization of the event. All AEs occurring while on study must be documented appropriately regardless of relationship and will be reported to JCCC DSMB / IRB.

Any medical condition that is present at the time that the participant is screened will be considered as baseline and not reported as an AE. However, if the study participant's condition deteriorates at any time during the study, it will be recorded as an AE.

Changes in the severity of an AE will be documented to allow an assessment of the duration of the event at each level of severity to be performed. AEs characterized as intermittent require documentation of onset and duration of each episode.

Nuclear medicine research coordinators will record all reportable events.

1.1.2 ADVERSE EVENT REPORTING

We do not anticipate hazardous situations for the subjects as a result of this protocol. However, procedures will be in place for verification of correct radiopharmaceutical dose and route of administration (i.e., each dose will be double checked for dosimetry and quality by a researcher and technologist). The study Principal Investigator (PI) or his designee will follow local IRB policy and 21 CFR 312.32 for adverse event reporting. If the principal investigator determines the unanticipated adverse device effect presents an unreasonable risk to subjects, the study will be terminated as soon as possible.

Adverse Event Reporting to IRB

All adverse events that meet the definition of an unanticipated problem will be reported to JCCC DSMB / IRB:
Within 10 working days:

- Any internal or external adverse event (unanticipated problem) which meets the following criteria:
  - Unexpected,
  - Related or possibly related to the research participation, and
  - Places subjects or others at greater risk of harm than was previously known or recognized (i.e. a serious adverse event, a new or increased risk to subjects/others)
- Internal adverse events that are expected and related but indicate a higher frequency of occurrence or a higher level of severity (i.e., indicating a new trend) that was not previously known and/or described in the approved informed consent document or other protocol related documents.
- External adverse event reports that indicate a potential risk which requires notification of previously enrolled subjects (i.e., malignancy), even if all subjects at the UCLA site have completed study participation.
- Any event that requires prompt reporting according to the IRB approved protocol or the study sponsor.

Within 2 working days:

- Internal subject deaths that meet the following criteria:

    - Occurred in an interventional study (i.e., involving a drug, biologic, device procedure and/or behavioral interventions),
    - Unexpected, and
    - Related or possibly related to research participation.

At Continuing Review:

- Internal subject deaths that meet the following criteria:
    - Occurred in an interventional study (i.e. involving a drug, biologic, device procedure and/or behavioral interventions),
    - Expected, and
    - Related or possibly related to research participation

Adverse Event Reporting to FDA

According to 21 CFR 312.32, the following events will be reported to the FDA:

15 day report:

- Serious and unexpected suspected adverse reaction (only if evidence to suggest a causal relationship between the drug and the adverse event)
- A single occurrence of an event that is not commonly and known to be strongly associated with drug exposure.
- One or more occurrences of an event that is not commonly associated with drug exposure, but is otherwise uncommon in the population exposed to the drug
- An aggregate analysis of specific events observed in a clinical trial (such as known consequences of the underlying disease or condition under investigation or other events that commonly occur in the study population independent of drug therapy) that indicates those events occur more frequently in the drug treatment group than in a concurrent or historical control group.
- Findings from other studies that suggest a significant risk in humans exposed to the drug.
- Findings from animal or in vitro testing that suggest a significant risk in humans exposed to the drug
- Increased rate of occurrence of serious suspected adverse reactions.

7 day report:

- Unexpected fatal or life-threatening suspected adverse reaction

1.1.3 SERIOUS ADVERSE EVENT REPORTING

Please refer to section 8.3.5 Adverse Event Reporting

2 STATISTICAL CONSIDERATIONS

2.1 STATISTICAL HYPOTHESES

We hypothesized that the per-patient detection rate of PSMA PET will be superior by 12% or more compared to bone scan + CT (35% vs 23%). The statistical test used to test our hypothesis was McNemar test. The level of the test was calculated using the standard alpha level of 0.05 which was 2 tailed.

2.2 SAMPLE SIZE DETERMINATION

A sample size of 102 patients achieves 80.3% power to detect a difference between two paired proportions of 0.12 which occurs when the proportion in cell 1,2 is 0.15 and the proportion in cell 2,1 is 0.03. The proportion of discordant pairs is 0.18. The contingency table numbers were obtained on the basis of:

- Literature: 18% overall positivity of BS + CT for M1b disease [8]
- Literature: 24% detection rate of PSMA-PET/CT in negative M1b disease on BS + CT [3]
- Clinical experience: 90% detection rate of PSMA-PET/CT in positive M1b disease on BS + CT

		**^{99m}Tc-MDP BS + CT**	
**^{68}Ga-PSMA PET/CT**		**+**	**-**
	**+**	**20 %**	**15 %**
	**-**	**3 %**	**61 %**

2.3 STATISTICAL ANALYSES

The detection rate of PSMA PET and bone scan + CT will be compared using the two-sided McNemar's test for paired proportions.

2.3.1 GENERAL APPROACH

Patient characteristics (e.g. age, PSA, Gleason) and study variables will be summarized with mean (SD), median (Q1-Q3) for continuous variables and frequency (%) for categorical variables. P-value ≤ .05 will be considered significant.

2.3.2 ANALYSIS OF THE PRIMARY EFFICACY ENDPOINT(S)

Primary Objective:

To compare the per-patient detection rate of ^{68}Ga-PSMA-11 PET/CT versus BS/CT for M1b disease in prostate cancer patients progressing after ADT. Patients will be treated as binary categorization as it follows: (i) Patients who have ≥1 positive bone lesion will be rated positive. (ii) Patients with no bone lesion detected will be rated as negative. The

analysis of the primary objective will utilize the McNemar's test to compare the detection rate between the imaging techniques.

2.3.3 ANALYSIS OF THE SECONDARY ENDPOINT(S)

Secondary Objectives:

- The number of lesions found by PSMA PET and bone scan + CT will be compared using a paired samples t-test.
- To compare the rate of M1 detection between the two modalities, defined as the number of patients with positive finding(s) for M1 disease (M1a or M1b or M1c) we will utilize the McNemar's test for paired proportions.
- To obtain per-patient PPV estimates (with 95% CI), defined as the number true positive findings the total number of positive cases (TP + FP). The reference standard will be based on information from procedures performed as per standard of care to the discretion of the referring physician: biopsy follow-up imaging, or changes in PSA levels. This information will only be available in a small subset of patients.
- To compare the prognostic value of PSMA PET versus bone scan derived tumor burden parameters
    - PSMA PET parameters: Total body PSMA tumor volume (PSMA-VOL) and SUVmean
    - Bone scan parameter: Bone scan index
    - Outcome data: PSA progression-free survival and Overall Survival

  In order to assess the prognostic ability of PSMA and Bone scan parameters, univariate Cox proportional hazards models for both PFS and OS will be constructed with models containing various patient characteristics as well as PSMA PET parameters and bone scan parameters (after we accrue approximately 4 years of follow-up data which we expect roughly 80% and 60% number of patients to have progressed or deceased). Hazard ratios with 95% confidence intervals as well as p-values will be tabulated. Next, we will explore multivariate models (using LASSO variable selection) including terms for important baseline characteristics as well as PSMA parameters or Bone scan parameters and we will extract the survival concordance indices from each of these models and compare them.

2.3.4 SAFETY ANALYSES

Adverse events and their severity will be reported as previously described in section 8.2 and 8.3.

2.3.5 PLANNED INTERIM ANALYSES

Not planned.

2.3.6 SUB-GROUP ANALYSES

We will construct risk groups using PSAdt to stratify the patients (<6 months or >6 months.) and run the same statistical analyses comparing the positivity rate between technologies to see if one of the technologies does better in more/less advanced patients.

2.3.7 TABULATION OF INDIVIDUAL PARTICIPANT DATA

2.3.8 EXPLORATORY ANALYSES

Not applicable

3 SUPPORTING DOCUMENTATION AND OPERATIONAL CONSIDERATIONS

3.1 REGULATORY, ETHICAL, AND STUDY OVERSIGHT CONSIDERATIONS

3.1.1 INFORMED CONSENT PROCESS

3.1.1.1 CONSENT/ASSENT AND OTHER INFORMATIONAL DOCUMENTS PROVIDED TO PARTICIPANTS

Consent forms describing in detail the study intervention, study procedures, and risks are given to the participant and written documentation of informed consent is required prior to starting intervention/administering study intervention.

3.1.1.2 CONSENT PROCEDURES AND DOCUMENTATION

Informed consent is a process that is initiated prior to the individual's agreeing to participate in the study and continues throughout the individual's study participation. Consent forms will be Institutional Review Board (IRB)-approved and the participant will be asked to read and review the document. The investigator will explain the research study to the participant and answer any questions that may arise. A verbal explanation will be provided in terms suited to the participant's comprehension of the purposes, procedures, and potential risks of the study and of their rights as research participants. Participants will have the opportunity to carefully review the written consent form and ask questions prior to signing. The participants should have the opportunity to discuss the study with their family or surrogates or think about it prior to agreeing to participate. The participant will sign the informed consent document prior to any procedures being done specifically for the study. Participants must be informed that participation is voluntary and that they may withdraw from the study at any time, without prejudice. A copy of the informed consent document will be given to the participants for their records. The informed consent process will be conducted and documented in the source document (including the date), and the form signed, before the participant undergoes any study-specific procedures. The rights and welfare of the participants will be protected by emphasizing to them that the quality of their medical care will not be adversely affected if they decline to participate in this study. Scan results from ^{68}Ga-PSMA-11 PET/CT will be provided to participants.

STUDY DISCONTINUATION AND CLOSURE

Not applicable.

3.1.2 CONFIDENTIALITY AND PRIVACY

Participant confidentiality and privacy is strictly held in trust by the participating investigators, their staff, and the sponsor(s) and their interventions. Therefore, the study protocol, documentation, data, and all other information generated will be held in strict confidence. No information concerning the study or the data will be released to any unauthorized third party without prior written approval of the sponsor.

All research activities will be conducted in as private a setting as possible.

The study participant's contact information will be securely stored at each clinical site for internal use during the study. At the end of the study, all records will continue to be kept in a secure location for as long a period as dictated by the reviewing IRB, Institutional policies, or sponsor requirements.

3.1.3 FUTURE USE OF STORED SPECIMENS AND DATA

Future use of the data not currently planned.

3.1.4 KEY ROLES AND STUDY GOVERNANCE

**Principal Investigators**	**Sponsor**
Jeremie Calais, MD;<br><br>Johannes Czernin, MD;<br><br>Matthias Benz, MD<br><br>Andrei Gafita, MD	Intramural funds
UCLA Nuclear Medicine	UCLA Nuclear Medicine
200 Medical Plaza Suite B114	200 Medical Plaza Suite B114
Los Angeles, CA 90095	Los Angeles, CA 90095
jcalais@mednet.ucla.edu<br>*jczernin@mednet.ucla.edu*<br><br>*mbenz@mednet.ucla.edu*<br><br>*agafita@mednet.ucla.edu*	*jczernin@mednet.ucla.edu*

3.1.5 SAFETY OVERSIGHT

Safety oversight is performed by Principle Investigators. In addition, it may subject to the oversight of UCLA Jonson Comprehensive Cancer Center (JCCC) Data and Safety Monitoring Board (DSMB).

3.1.6 CLINICAL MONITORING

UCLA DSMB have the oversight for trial conduct and compliance. This trial may subject to DSMB monitoring

3.1.7 QUALITY ASSURANCE AND QUALITY CONTROL

Not applicable.

3.1.8 DATA HANDLING AND RECORD KEEPING

3.1.8.1 DATA COLLECTION AND MANAGEMENT RESPONSIBILITIES

The CRFs will be stored in a locked office in the Nuclear Medicine Clinic.

During the clinical investigation, the Principal Investigator will evaluate the progress of the trial, including periodic assessments of data quality and timeliness, participant recruitment, accrual and retention, participant risk versus benefit, performance of trial sites, and other factors that can affect study outcome.

At the end of the study, source document will be digitally archived at an off-site HIPAA compliant digital storage site managed by UCLA Health IT.

3.1.8.2 STUDY RECORDS RETENTION

Study documents should be retained for a minimum of 2 years after the last approval of a marketing application in an International Conference on Harmonization (ICH) region and until there are no pending or contemplated marketing applications in an ICH region or until at least 2 years have elapsed since the formal discontinuation of clinical development of the study intervention. These documents should be retained for a longer period, however, if required by local regulations. No records will be destroyed without the written consent of the sponsor, if applicable. It is the responsibility of the sponsor to inform the investigator when these documents no longer need to be retained.

3.1.9 PROTOCOL DEVIATIONS

A protocol deviation is any noncompliance with the clinical trial protocol. The noncompliance may be either on the part of the participant, the investigator, or the study site staff. As a result of deviations, corrective actions are to be developed by the site and implemented promptly.

3.1.10 PUBLICATION AND DATA SHARING POLICY

Not applicable.

3.1.11 CONFLICT OF INTEREST POLICY

Not applicable.

3.2 ADDITIONAL CONSIDERATIONS

Not applicable.

3.3 ABBREVIATIONS

AE	Adverse Event
Bone scan	99mTc MDP bone scintigraphy
BS	99mTc MDP bone scintigraphy
CFR	Code of Federal Regulations
CRF	Case Report Form
CT	Computational Tomography
DSMB	Data Safety Monitoring Board
EOS	End of Synthesis
FDA	Food and Drug Administration
GCP	Good Clinical Practice
GLP	Good Laboratory Practices
HIPAA	Health Insurance Portability and Accountability Act
ICH	International Conference on Harmonisation
IND	Investigational New Drug Application
IRB	Institutional Review Board
JCCC	Jonson Comprehensive Cancer Center
M1a	Lymph node metastases
M1b	Bone metastases
M1c	Visceral metastases
PCa	Prostate Cancer
PET	Positron Emission Tomography
PSMA	Prostate Specific Membrane Antigen
PI	Principal Investigator
SAE	Serious Adverse Event
SOA	Schedule of Activities
UCLA	University of California Los Angeles
UP	Unanticipated Problem

3.4 PROTOCOL AMENDMENT HISTORY

Version	Date	Description of Change	Brief Rationale
1	01/13/2021<br>Rev 2/9/2021<br>Rev 5/21/2021	Original Protocol	
2	07/22/2021	Final report, FDA label, imaging protocol	Report requires more time to be finalized, PSMA scan approved by FDA, Fasting is required

11 REFERENCES

[1] Hofman MS, Lawrentschuk N, Francis RJ, Tang C, Vela I, Thomas P, et al. Prostate-specific membrane antigen PET-CT in patients with high-risk prostate cancer before curative-intent surgery or radiotherapy (proPSMA): a prospective, randomised, multicentre study. Lancet. 2020;395:1208-16.
[2] Fendler WP, Calais J, Eiber M, Flavell RR, Mishoe A, Feng FY, et al. Assessment of 68Ga-PSMA-11 PET Accuracy in Localizing Recurrent Prostate Cancer: A Prospective Single-Arm Clinical Trial. JAMA Oncol. 2019.
[3] Fendler WP, Weber M, Iravani A, Hofman MS, Calais J, Czernin J, et al. Prostate-Specific Membrane Antigen Ligand Positron Emission Tomography in Men with Nonmetastatic Castration-Resistant Prostate Cancer. Clin Cancer Res. 2019.
[4] Weber M, Kurek CE, Barbato F, Eiber M, Maurer T, Nader M, et al. PSMA-ligand PET for early castration-resistant prostate cancer: a retrospective single-center study. J Nucl Med. 2020.
[5] Moreira DM, Howard LE, Sourbeer KN, Amarasekara HS, Chow LC, Cockrell DC, et al. Predicting bone scan positivity in non-metastatic castration-resistant prostate cancer. Prostate Cancer Prostatic Dis. 2015;18:333-7.
[6] https://www.fda.gov/media/76286/download.
[7] Eiber M, Herrmann K, Calais J, Hadaschik B, Giesel FL, Hartenbach M, et al. Prostate Cancer Molecular Imaging Standardized Evaluation (PROMISE): Proposed miTNM Classification for the Interpretation of PSMA-Ligand PET/CT. J Nucl Med. 2018;59:469-78.
[8] Smith MR, Saad F, Chowdhury S, Oudard S, Hadaschik BA, Graff JN, et al. Apalutamide Treatment and Metastasis-free Survival in Prostate Cancer. New England Journal of Medicine. 2018;378:1408-18.
