# Supplementary material for: Phase 2 trial of PSMA PET CT versus planar bone scan and CT in prostate cancer patients progressing while on androgen deprivation therapy
Source: Sci Rep. 2024 Oct 18;14:24411. doi: 10.1038/s41598-024-75589-6 (PMC11487247; doi:10.1038/s41598-024-75589-6)
Supplement: Supplementary file 4 — Supplementary Material 4. [file 41598_2024_75589_MOESM4_ESM.docx]

**Supplementary Table 1:** Contingency table illustrating the hypothesis for power analysis (planned *n* = 102 patients). This assumes a 35% detection rate on ^68^Ga-PSMA PET/CT and a 23% detection rate on ^99m^Tc-MDP bone scan plus CT.

|  | **^99m^Tc-MDP Bone Scan plus CT**  **+** | **^99m^Tc-MDP Bone Scan plus CT**  **-** |
| --- | --- | --- |
| **^68^Ga-PSMA PET/CT**  **+** | 20 % | 15 % |
| **^68^Ga-PSMA PET/CT**  **-** | 3 % | 61 % |

*Abbreviations: PSMA PET/CT = prostate-specific membrane antigen positron emission tomography/computed tomography.*

**Supplemental Table 2.** Pairwise agreement of the six individual independent central readers evaluating the presence of bone metastasis. Measures of agreement were calculated using Kappa coefficient with 95% confidence intervals (CIs).

|  | **^68^Ga-PSMA PET/CT** | | | **^99m^Tc-MDP Bone Scan plus CT** | | |
| --- | --- | --- | --- | --- | --- | --- |
|  | **Reader 1 vs 2** | **Reader 1 vs 3** | **Reader 2 v 3** | **Reader 4 vs 5** | **Reader 4 vs 6** | **Reader 5 v 6** |
| **Kappa coefficient** | 0.89 | 1 | 0.89 | 0.58 | 0.89 | 0.67 |
| **95% CI** | 0.68-1 | N/A | 0.68-1 | 0.21-0.95 | 0.68-1 | 0.33-1 |

*Abbreviations: PSMA PET/CT = prostate-specific membrane antigen positron emission tomography/computed tomography.*

**Supplemental Table 3.** Per-patient reads of the three independent central readers (*n* = 22 patients) evaluating the detection of extra-osseous metastasis on PSMA PET/CT.

|  | **T+ on ^68^Ga-PSMA PET/CT** | | | **N+ on ^68^Ga-PSMA PET/CT** | | | **M1a on ^68^Ga-PSMA PET/CT** | | | **M1c on ^68^Ga-PSMA PET/CT** | | |
| --- | --- | --- | --- | --- | --- | --- | --- | --- | --- | --- | --- | --- |
| **Patient No.** | **Reader 1** | **Reader 2** | **Reader 3** | **Reader 1** | **Reader 2** | **Reader 3** | **Reader 1** | **Reader 2** | **Reader 3** | **Reader 1** | **Reader 2** | **Reader 3** |
| **1** | **Yes** | **Yes** | **Yes** | **Yes** | No | No | **Yes** | **Yes** | **Yes** | No | No | No |
| **2** | **Yes** | **Yes** | **Yes** | No | No | No | No | No | No | No | No | No |
| **3** | No | No | No | No | No | No | No | No | No | No | **Yes** | No |
| **4** | No | No | No | No | No | No | **Yes** | **Yes** | No | No | No | No |
| **5** | No | No | No | **Yes** | No | **Yes** | **Yes** | No | **Yes** | No | No | No |
| **6** | **Yes** | **Yes** | **Yes** | **Yes** | No | No | No | No | No | No | No | No |
| **7** | No | No | No | No | No | No | No | No | No | No | No | No |
| **8** | **Yes** | No | **Yes** | **Yes** | **Yes** | **Yes** | No | No | **Yes** | No | No | No |
| **9** | No | No | No | No | No | No | No | No | No | **Yes** | **Yes** | **Yes** |
| **10** | No | No | No | No | No | No | No | No | No | No | No | No |
| **11** | No | No | No | No | No | No | No | No | No | No | No | No |
| **12** | No | No | No | No | No | No | No | No | No | No | No | No |
| **13** | No | No | No | No | No | No | No | No | **Yes** | No | No | No |
| **14** | No | No | No | No | No | No | No | No | No | No | No | No |
| **15** | **Yes** | **Yes** | **Yes** | No | No | No | No | No | No | No | No | No |
| **16** | **Yes** | **Yes** | **Yes** | **Yes** | **Yes** | **Yes** | No | No | No | No | No | No |
| **17** | **Yes** | **Yes** | **Yes** | No | No | No | No | No | No | No | No | No |
| **18** | No | **Yes** | No | No | No | No | No | No | No | No | No | No |
| **19** | **Yes** | **Yes** | **Yes** | No | No | No | No | No | No | No | No | No |
| **20** | No | No | No | No | No | No | No | No | No | No | No | No |
| **21** | No | No | No | **Yes** | **Yes** | No | No | No | No | No | No | No |
| **22** | **Yes** | **Yes** | **Yes** | **Yes** | **Yes** | **Yes** | **Yes** | **Yes** | **Yes** | **Yes** | **Yes** | **Yes** |
| **Total** | **9/22** | **9/22** | **9/22** | **7/22** | **4/22** | **4/22** | **4/22** | **3/22** | **5/22** | **2/22** | **3/22** | **2/22** |
| *Abbreviations: PSMA PET/CT = prostate-specific membrane antigen positron emission tomography/computed tomography.* | | | | | | | | | | | | |

**Supplemental Table 4:** Per-patient reads of the three independent central readers (*n* = 22 patients) evaluating the detection of extra-osseous metastasis on the CT portion of the PET/CT.

|  | **T+ on CT**  **(≥10 mm)** | | | **N+ on CT**  **(≥15 mm)** | | | **M1a on CT**  **(≥15 mm)** | | | **M1c on CT**  **(≥10 mm)** | | |
| --- | --- | --- | --- | --- | --- | --- | --- | --- | --- | --- | --- | --- |
| **Patient No.** | **Reader 1** | **Reader 2** | **Reader 3** | **Reader 1** | **Reader 2** | **Reader 3** | **Reader 1** | **Reader 2** | **Reader 3** | **Reader 1** | **Reader 2** | **Reader 3** |
| **1** | No | No | No | No | No | No | No | **Yes** | **Yes** | No | No | **Yes** |
| **2** | No | No | No | No | No | No | No | No | No | No | No | No |
| **3** | No | No | No | No | No | No | No | No | No | **Yes** | No | No |
| **4** | No | No | No | No | No | No | No | No | No | No | No | No |
| **5** | No | No | No | No | No | **Yes** | No | No | **Yes** | No | No | No |
| **6** | No | No | No | No | No | No | No | No | No | No | No | No |
| **7** | No | No | No | No | No | No | No | No | No | No | No | No |
| **8** | **Yes** | **Yes** | **Yes** | **Yes** | **Yes** | **Yes** | No | No | No | No | No | No |
| **9** | No | No | No | No | No | No | No | No | No | No | No | No |
| **10** | No | No | No | No | No | No | No | No | No | No | No | No |
| **11** | No | No | No | No | No | No | No | No | No | No | No | No |
| **12** | No | No | No | No | No | No | No | No | No | No | No | No |
| **13** | No | No | No | No | No | **Yes** | No | No | **Yes** | No | No | No |
| **14** | No | No | No | No | No | No | No | No | No | No | No | No |
| **15** | No | Yes | No | No | No | No | No | No | No | No | No | No |
| **16** | **Yes** | **Yes** | **Yes** | No | **Yes** | No | No | No | No | No | No | No |
| **17** | No | **Yes** | No | No | No | No | No | No | No | No | No | No |
| **18** | No | No | No | No | No | No | No | No | No | No | No | No |
| **19** | **Yes** | **Yes** | **Yes** | No | No | No | No | No | No | No | No | No |
| **20** | No | No | No | No | No | No | No | No | No | No | No | No |
| **21** | No | No | No | **Yes** | **Yes** | No | No | No | No | No | No | No |
| **22** | No | **Yes** | No | **Yes** | **Yes** | **Yes** | **Yes** | **Yes** | **Yes** | **Yes** | **Yes** | **Yes** |
| **Total** | **3/22** | **6/22** | **3/22** | **3/22** | **4/22** | **4/22** | **1/22** | **2/22** | **4/22** | **2/22** | **1/22** | **2/22** |

*Abbreviations: PSMA PET/CT = prostate-specific membrane antigen positron emission tomography/computed tomography.*

|  | **CT Alone**  **+** | **CT Alone**  **-** |
| --- | --- | --- |
| **^68^Ga-PSMA PET/CT**  **+** | **27 %** | **32 %** |
| **^68^Ga-PSMA PET/CT**  **-** | **0 %** | **41 %** |

**Supplemental Table 5:** Contingency table of the consensus majority reads per patient (*n* = 22 patients) comparing the detection rates of extra-osseous metastasis by ^68^Ga-PSMA PET/CT versus CT alone.

*Abbreviations: PSMA PET/CT = prostate-specific membrane antigen positron emission tomography/computed tomography.*

**Supplemental Table 6.** Pairwise agreement of the three individual independent central readers evaluating the presence of extra-osseous metastasis. Measures of agreement were calculated using Kappa coefficient with 95% confidence intervals (CIs).

|  | **^68^Ga-PSMA PET/CT** | | | **CT Alone** | | |
| --- | --- | --- | --- | --- | --- | --- |
|  | **Reader 1 vs 2** | **Reader 1 vs 3** | **Reader 2 v 3** | **Reader 1 vs 2** | **Reader 1 vs 3** | **Reader 2 v 3** |
| **Kappa coefficient** | 0.71 | 0.72 | 0.44 | 0.59 | 0.46 | 0.50 |
| **95% CI** | 0.41-1 | 0.43-1 | 0.07-0.81 | 0.23-0.94 | 0.05-0.86 | 0.12-0.88 |

*Abbreviations: PSMA PET/CT = prostate-specific membrane antigen positron emission tomography/computed tomography.*
